# Visiting the Sick

**Published:** 1875-04

**Authors:** 


### VISITING THE SICK.

To visit the sick is a dictate of humanity, a scriptural injunction. But we devoutly wish that Paul or Peter, or John, had in one of his epistles laid down the precept thus: "Visit the sick, but make *short visits.*" With no irreverence, we may say that had either of these saints known how few people are fit to enter a sick-room he would not have written any rule in reference to such duty without that important qualification.

We may say without much exaggeration, that visitors kill more than the doctors. Many a physician has found the result of his most skillful treatment, and the watchful care of an intelligent nurse, destroyed by some fool of a visitor who in an unfortunate moment has gained access to the sick-room. The patient was doing well; she had passed the critical line of the disease, and was on the road to recovery. But she was still feeble and unable to bear the least excitement or fatigue. At this time Mrs. Gabbleton calls. The family do not want to hurt Mrs. G.'s feelings by refusing to permit her to see the sick lady, and so she is admitted. She tarries three hours, and is "very entertaining;" that is, she talks incessantly. The poor patient is worried and exhausted; she passes a restless night, and, when the doctor makes his morning call, he finds a quick pulse; dry, hot skin, and a returned fever. He has no difficulty in accounting for this unfavorable change, as soon as he learns what has occurred. He "blesses" Mrs. Gabbleton, and those who risked the life of his patient rather than injure the woman's feelings.

The "moral" is obvious: *beware of visitors.* Do not let any nice points of etiquette, or tender regard for the visitor, stand in the way of proper care for the patient. It is better to be too cautious, and err on the safe side, by keeping the patient secluded longer than is really necessary. The physician can not be too explicit in his orders in this respect.

---

Any lady who keeps house-plants knows enough to keep them where they will get the benefit of sunlight. You may look at the lilies, roses, pinks, and dahlias, of your flower-garden, and you will notice they all have beautiful colors. You may rear those same flowers in places where the sunlight is entirely excluded, and keep them in the dark, or supply them with artificial light only, such as candles, lamps, and gaslight, and you will find they will not have that pure, brilliant color, which nature designed they should have. The same is true of men, women, and children, if they are deprived of sunlight. Demonstrations of this fact may be seen in your fashionable parlors, where women and children spend most of their time, and the windows are kept blinded to keep out the sunlight for fear it might injure the carpet.

---

How great a delight is good health to all creatures!

